# The Utility of Ground Bryophytes in the Assessment of Soil Condition in Heavy Metal-Polluted Grasslands

**DOI:** 10.3390/plants11162091

**Published:** 2022-08-11

**Authors:** Kaja Rola, Vítězslav Plášek

**Affiliations:** 1 Institute of Botany, Faculty of Biology, Jagiellonian University, Gronostajowa 3, 30-387 Kraków, Poland; 2 Department of Biology and Ecology, Faculty of Science, University of Ostrava, Chittussiho 10, CZ-710 00 Ostrava, Czech Republic; 3 Institute of Biology, University of Opole, Oleska 22, 45-052 Opole, Poland

**Keywords:** bryophytes, bioindication, heavy metals, soil pH, soil pollution, species richness

## Abstract

Bryophytes are commonly used in biomonitoring heavy metal pollution, whereas the bioindicative value of bryophyte communities is a less known issue. The aim of the present study is to recognize the utility of the bryophyte community’s structure in the assessment of soil condition in heavy metal-polluted, dry grasslands. The study plots are examined with respect to bryophytes; vascular plants; concentrations of Zn, Pb, Cd, and As in the soil; total nitrogen and organic carbon content in the soil; and soil pH. The results show that both bryophyte species richness and composition greatly depend on soil chemical characteristics, including heavy-metal pollution levels and soil pH. Three groups of species are distinguished: (1) species sensitive to pollution growing on acidic soils, (2) nonspecific species inhabiting a wide spectrum of heavy metal-polluted sites, and (3) species preferring polluted and alkaline soils. Our study reveals a gradual replacement of the bryophyte species alongside increasing soil pollution and alkalinity. This proves that bryophytes are highly responsive to soil factors and the changes in bryophyte composition may indicate the soil condition of a certain site. Furthermore, high concentrations of heavy metals in the soil and an alkaline pH positively affect bryophyte species richness. Consequently, such sites could be considered as biodiversity hotspots for terrestrial bryophytes in post-industrial landscapes.

## 1. Introduction

Bryophytes are an informal group of three divisions of non-vascular land plants, which consists of about 25,000 species known worldwide [1]. They play an essential role in several terrestrial ecosystems and their relevance is particularly pronounced in polar, mountain, and nutrient-poor grassland areas, where they frequently dominate over vascular plants in terms of their abundance and biomass [2,3]. These organisms are also known as essential and rapid colonizers of bare ground in the context of the succession of both natural and anthropogenic habitats [4,5]. In contrast to vascular plants, bryophytes lack root systems, protective cuticles, or other filtration mechanisms [6], which could prevent the passive import of toxic substances and heavy metals into their cells [7,8]. Due to their physiological nature, many bryophytes are sensitive to pollution, which makes them highly responsive to spatial and temporal fluctuations of diverse pollutants in the surrounding environment. For this reason, they have been extensively used to monitor the level of heavy metal elements [9].

Anthropogenic habitat disturbances are important drivers of change in both biodiversity and species composition worldwide. Heavy metal pollution generally leads to a decrease in biodiversity and the impoverishment of bryophyte communities [10]. On the other hand, cryptogamic organisms have been defined as stress tolerant [11], and some bryophyte species have been proved to occur abundantly on metal-rich substrates (e.g., [5,12,13,14]). Moreover, their occurrence in polluted sites is frequently favored by minor competition from vascular plants [11,15]. Nevertheless, the differentiated responses of particular species has led to changes in the structure and species composition of bryophyte communities in the vicinity of pollution sources [14,16]. The use of bryophytes for biomonitoring purposes by measuring heavy metal concentrations in these organisms is a much more frequently studied issue, whereas the bioindicative value of bryophyte assemblages expressed by the changes in species composition reflecting the level of soil pollution is a less known aspect [13]. Recently, a global meta-analysis on the impact of industrial polluters on bryophytes has been conducted [14]; however, the analysis concerns the effects of point polluters that affect bryophyte communities primarily via the ambient air in the impact zone. The present study is related to the direct effects of metal elements accumulated on the soil substrate along with other basic soil chemical parameters that could potentially affect ground bryophyte communities.

The main aim of the present study is to recognize the changes in bryophyte species composition, richness, and abundance in relation to soil chemical characteristics at heavy metal-polluted sites. We also aim to determine the preferences of particular bryophyte species in relation to soil chemical parameters and to estimate the indicator value of bryophyte community structure and bryophyte species richness for the assessment of soil conditions.

## 2. Results

### 2.1. Soil Properties of Heavy Metal-Polluted Sites

**Figure 1:**
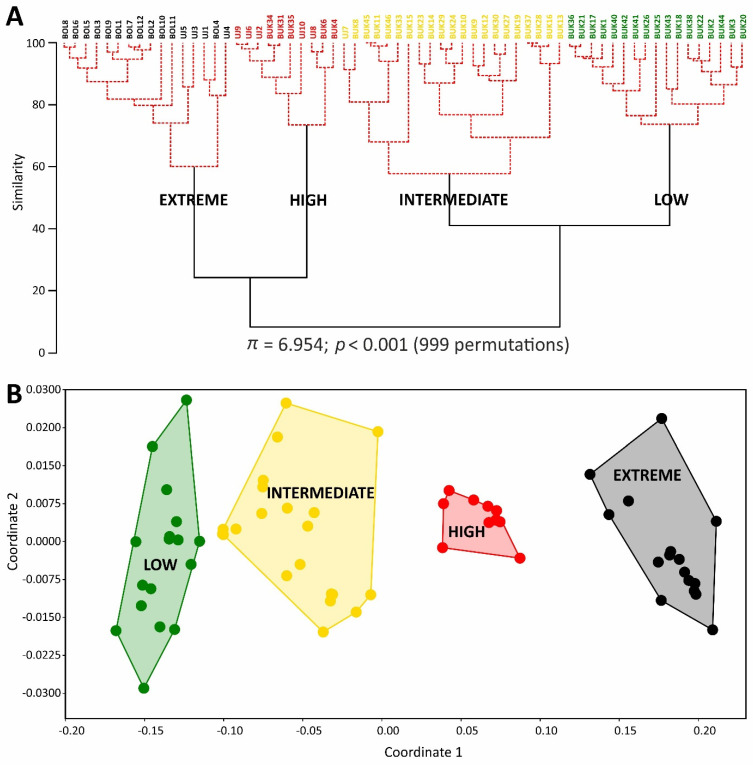
Cluster analysis dendrogram (**A**) and non-metric multidimensional scaling (NMDS) scatterplot (**B**) of study plots assigned to four soil condition classes. The black lines in the dendrogram indicate groups that are established; red lines show a sub-structure from the clustering for which there is no statistical support from the SIMPROF test. Convex hulls on the NMDS scatterplot encompass particular soil condition classes.

Cluster analysis and an NMDS diagram revealed consistent results, and both separated the study plots into four groups (Figure 1). The similarity profile test (SIMPROF) confirmed the significance of the designated groups (π = 6.954; *p* < 0.001). Consequently, four soil condition classes were identified, i.e., ‘low’, ‘intermediate’, ‘high’, and ‘extreme’.

**Figure 2:**
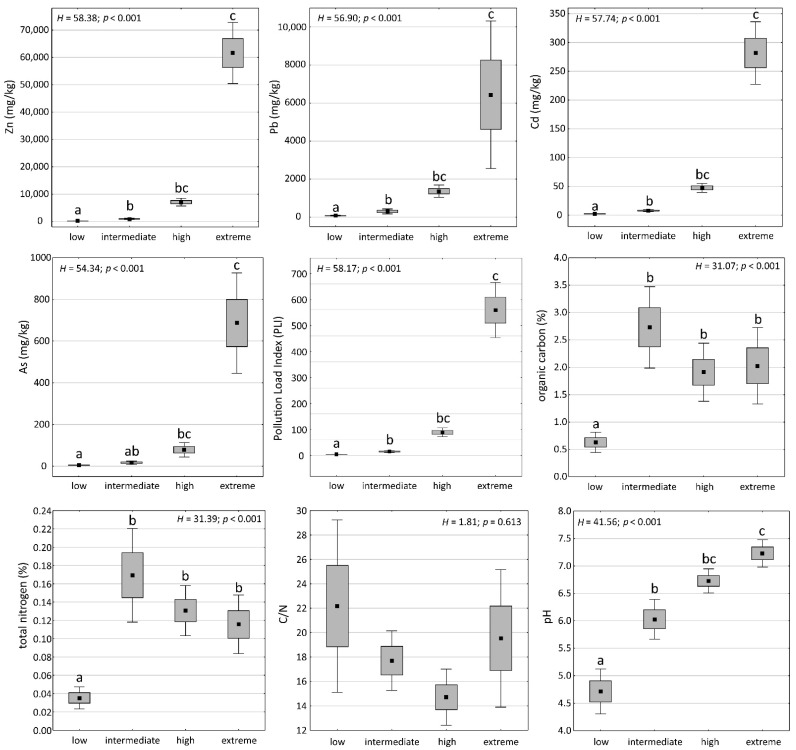
Box-and-whisker plots of soil chemical parameters and pollution load index (PLI) for particular soil condition classes. The points indicate means, boxes indicate standard errors, and whiskers 95% confidence intervals. The results of Kruskal–Wallis test (*p* < 0.05) are included: H and *p*-values. The letters denote the results of Dunn’s post hoc test; different letters indicate significant differences at the *p* < 0.05 level.

According to the Kruskal–Wallis test (*p* < 0.05), the distinguished soil condition classes differed significantly in terms of soil chemical properties (Figure 2). The exception was the C/N ratio for which no significant differences between the soil condition classes were recorded (*p* > 0.05). The differences in heavy metal concentrations were the most prominent. Zn, Pb, Cd, and As concentrations were the highest in the ‘extreme’ class and significantly differed from the ‘low’ and ‘intermediate’ classes. The same issues concerned the pollution load index (PLI). As regards organic carbon and total nitrogen, the ‘low’ class was characterized by significantly lower contents of these elements compared to the remaining classes. The soil pH showed an upward trend along with increasing soil pollution, trending from very strongly acidic in the ‘low’ pollution class through to slightly acidic and neutral up to slightly alkaline in the ‘extreme’ class (Figure 2).

### 2.2. Bryophyte Species Richness and Cover in Soil Condition Classes

**Figure 3:**
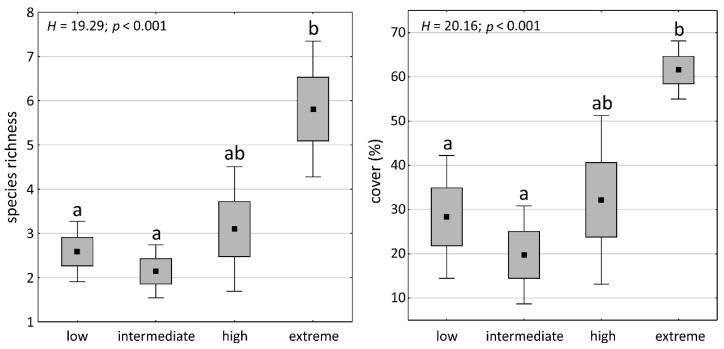
Box-and-whisker plots of bryophyte species richness and cover in particular soil condition classes. The points indicate the means, boxes indicate standard errors, and whiskers 95% confidence intervals. The results of Kruskal–Wallis test (*p* < 0.05) are included: H and *p*-values. The letters denote the results of Dunn’s post hoc test; different letters indicate significant differences at the *p* < 0.05 level.

Altogether, 32 bryophyte species from 22 genera were recorded. More specifically, 1 species of liverwort, 16 species of acrocarpous mosses, and 15 species of pleurocarpous mosses were identified. Among the acrocarpous mosses, species of Bryaceae and Pottiaceae families dominated. On the contrary, Brachytheciaceae was the most frequently represented family among pleurocarpous mosses. Species richness differed between soil condition classes, being significantly higher in the ‘extreme’ class than in the ‘low’ and ‘intermediate’ classes (Kruskal–Wallis test; *p* < 0.05). The same concerned the total bryophyte cover (Figure 3).

Principal component analysis based on soil chemical characteristics showed a clear distribution of all four soil condition classes along the first axis (Figure S1). The most distinctive was the ‘extreme’ class isolated on the right side of the PCA diagram, which was simultaneously characterized by usually higher numbers of bryophyte species recorded in the study plots.

### 2.3. Factors Affecting Bryophyte Species Richness and Cover

The factor analysis reduced 9 variables to 4 factors that jointly explained 90.67% of the total variation (Table 1). Factor 1 was negatively related to vascular plant cover. Factor 2 was associated with organic carbon and total nitrogen contents in the soil and accordingly corresponded to soil fertility. Factor 3 was related to heavy metal concentrations in the soil and PLI, and thus could be referred to soil pollution. Factor 4 was associated with the soil pH.

The results of the multiple stepwise regression analysis are presented in Table 2. The forward stepwise procedure with 4 predictors (factors) and bryophyte species richness as the dependent variable revealed that only three factors were included in the model (F = 17.70, *p* < 0.05). All of them showed significant effects. Factor 1 negatively related to vascular plant cover positively influenced species richness of bryophytes. This factor exerted the greatest influence and may be considered the most important predictor based on the standardized β coefficient. Subsequently, factor 2 related to soil pollution had a positive effect on species richness. The last significant factor was factor 4 related to soil pH. As regards bryophyte cover, the same three factors were also included in the model (F = 17.70, *p* < 0.05). Two of them, i.e., factors 1 and 3, showed a significant effect on bryophyte cover. Factor 4, although included in the model, had no significant effect on bryophyte cover (*p* > 0.05).

### 2.4. Bryophyte Species Composition

PERMANOVA results show that species composition significantly differs between the plots representing different soil condition classes (F = 7.44; *p* < 0.001). Pairwise comparisons between classes showed that each class significantly differed from each other (Table 3). The similarity in the species composition between particular classes was not greater than 30%. Moreover, a significant correlation between bryophyte species composition similarity and soil chemical parameter similarity was confirmed by the Mantel test (R = 0.40, *p* < 0.05). This indicated that the similarity of species composition of bryophyte communities increased with the increasing similarity of soil chemical parameters.

**Figure 4:**
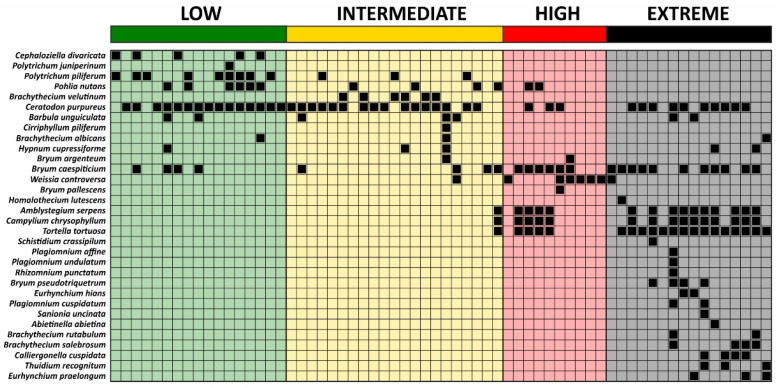
Seriation diagram based on the constrained algorithm of species occurrence in the examined plots arranged according to soil condition classes. For details on soil condition classes, see Figure 1 and Figure 2.

The seriation diagram shows the pattern of bryophyte species occurrences across particular soil condition classes (Figure 4). The species tend to spread along diagonally, which indicates a gradual change in the species composition along with soil acidity and pollution level. The ‘low’ class was characterized by the presence of species exclusive to this class, i.e., Cephaloziella divaricata and *Polytrichum juniperinum*, species occurring frequently in this class and also in the ‘intermediate’ class, i.e., *Polytrichum piliferum*, as well as nonspecific species, which have a broad spectrum and appear in most soil condition classes. The ‘intermediate’ class showed a similar pattern; two exclusive species were recorded, i.e., Brachythecium velutinum and Cirriphyllum piliferum. In the ‘high’ class, species present in various soil condition classes dominated; additionally, a few species, i.e., Weissia controversa, Amblystegium serpens, Campylium chrysophyllum, and *Tortella tortuosa*, found only in this class and the ‘extreme’ class were recorded. Bryum pallescens was the only species exclusive to this class. The ‘extreme’ class was characterized by the highest number of exclusive species (Figure 4).

**Figure 5:**
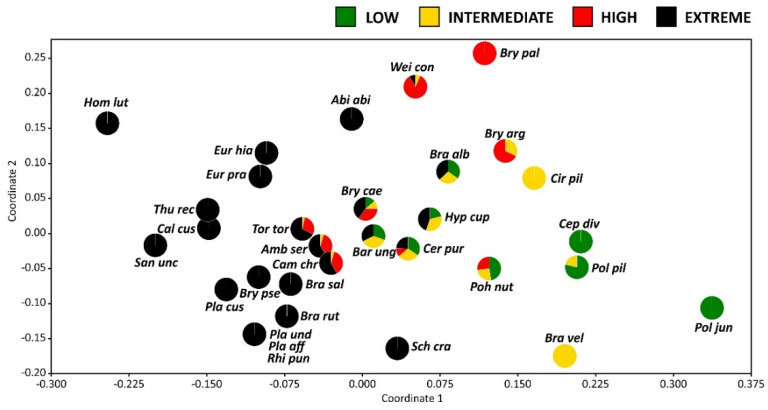
Non-metric multidimensional scaling (NMDS) scatterplot presenting similarities in terms of bryophyte species occurrence in the studied plots. The pie charts present the proportions of the occurrence of a given species in particular soil condition classes (based on a matrix of the frequency of a species in a given class). The abbreviations for the species are as follows: *Abi abi*—*Abietinella abietina*; *Amb ser*—*Amblystegium serpens*; *Bar ung*—*Barbula unguiculata*; *Bra alb*—*Brachythecium albicans*; *Bra vel*—*Brachythecium velutinum*; *Bry arg*—*Bryum argenteum*; *Bry cae*—*Bryum caespiticium*; *Bry pal*—*Bryum pallescens*; *Bry pse*—*Bryum pseudotriquetrum*; *Bra rut*—*Brachythecium rutabulum*; *Bra sal*—*Brachythecium salebrosum*; *Cal cus*—*Calliergonella cuspidata*; *Cam chr*—*Campylium chrysophyllum*; *Cep div*—*Cephaloziella divaricata*; *Cer pur*—*Ceratodon purpureus*; *Cir pil*—*Cirriphyllum piliferum*; *Eur hia*—*Eurhynchium hians*; *Eur pra*—*Eurhynchium praelongum*; *Hom lut*—*Homalothecium lutescens*; *Hyp cup*—*Hypnum cupressiforme*; *Pla aff*—*Plagiomnium affine*; *Pla cus*—*Plagiomnium cuspidatum*; *Pla und*—*Plagiomnium undulatum*; *Poh nut*—*Pohlia nutans*; *Pol jun*—*Polytrichum juniperinum*; *Pol pil*—*Polytrichum piliferum*; *Rhi pun*—*Rhizomnium punctatum*; *San unc*—*Sanionia uncinata*; *Sch cra*—*Schistidium crassipilum*; *Thu rec*—*Thuidium recognitum*; *Tor tor*—*Tortella tortuosa*; *Wei con*—*Weissia controversa*.

The NMDS ordination diagram visualized the similarities between bryophyte species based on their occurrence in the studied plots (Figure 5). The species co-occurrence pattern along the first axis could be fairly related to the soil condition gradient. Species concentrated on the left side of the diagram were restricted to the presence in plots representing the ‘extreme’ class. Conversely, species positioned on the right-hand side were primarily associated with the ‘low’ class. The species indifferent to heavy metal pollution and soil pH were grouped in the central part of the diagram between the two aforementioned groups. They proved to occur more or less equally often in all soil condition classes.

**Figure 6:**
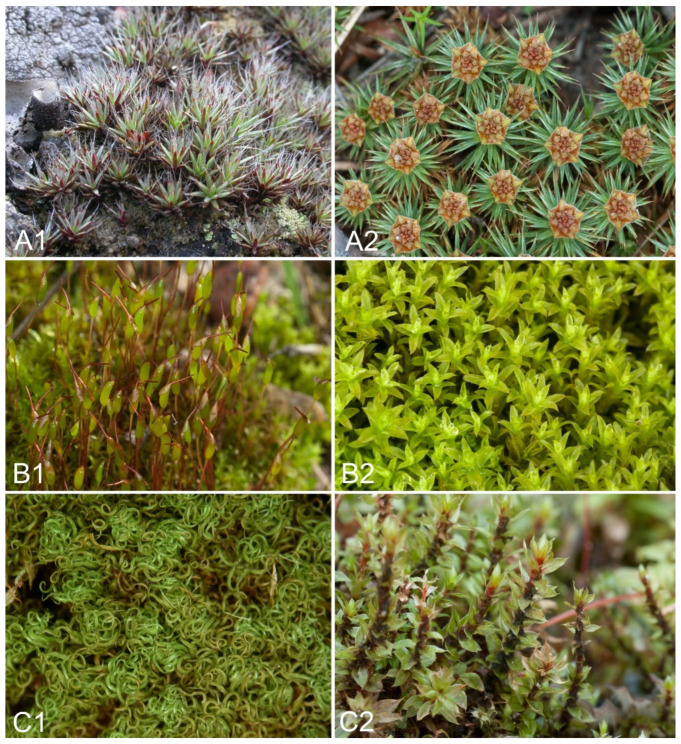
Examples of bryophyte species representing the three defined groups (**A**–**C**) according to their sensitivity to heavy metal pollution. (**Group A**): (**A1**)—*Polytrichum piliferum*, (**A2**)—*Polytrichum juniperinum*; (**Group B**): (**B1**)—*Ceratodon purpureus*, (**B2**)—*Barbula unguiculata*; (**Group C**): (**C1**)—*Tortella tortuosa*, (**C2**)—*Bryum pseudotriquetrum*.

Based on the results above and the overall relative frequency of each bryophyte species, the following classification of bryophytes into three different groups of species was proposed (Table 4; Figure 6).

### 2.5. Ecological Preferences of Bryophytes 

**Figure 7:**
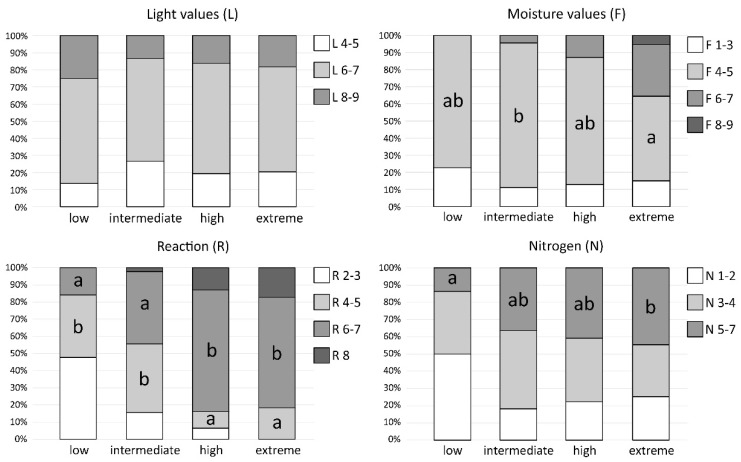
Composition of bryophytes in particular soil condition classes in the context of habitat requirements according to their ecological indicator values: light (L), moisture (F), reaction (acidity) (R), and nitrogen (eutrophication) (N) values. The results of Dunn’s post hoc test are provided when the Kruskal–Wallis test indicated significant differences between the soil condition classes; different letters within the bars indicate significant differences at the *p* < 0.05 level.

Bryophyte species composition in particular soil condition classes significantly differed in terms of the participation of the bryophyte species with ecological preferences to moisture, pH, and nitrogen (Figure 7, Table S1). As regards the proportion of bryophytes classified into the ranges of light values (L), no significant differences between soil condition classes were observed. With regard to the moisture preferences of the species, the proportion of bryophytes considered as dry-site indicators (range 1–3) also did not differed between soil condition classes, whereas the share of bryophytes associated with well-drained and moderately moist terrestrial substrata (range 4–5) was significantly higher in the ‘intermediate’ than in the ‘extreme’ class. The proportion of bryophytes growing on moist/constantly moist substrata (range 6–7) increased along with the increasing soil pH and pollution. The species representing the range of 8–9 were recorded only in the ‘extreme’ class. The most distinct trend was observed for changes in the share of bryophytes preferring a different soil pH. The ‘high’ and ‘extreme’ classes were characterized by significantly higher proportions of bryophytes associated with basic and strongly basic substrata (range 6–7) and a significantly lower proportion of bryophytes preferring acid and moderately acid soils (range 4–5) than the ‘low’ and ‘intermediate’ classes. In the ‘extreme’ class, species confined to acid substrata were not observed (range 2–3). Conversely, species associated with strongly basic substrata (indicator value of 8) were not recorded in the ‘low’ class. As regards the ecological preferences of bryophytes to eutrophication, significant differences were observed in the proportion of species associated with moderately fertile and fertile substrata (range 5–7), being significantly lower in the ‘low’ than in the ‘extreme’ class.

## 3. Discussion

Bryophytes have developed various morphological, physiological, and reproductive adaptations, allowing them to survive in adverse habitat conditions [17,18]. Nevertheless, various studies have shown that bryophyte species differ considerably in their sensitivity to different pollutants [4,13,19,20]. Some species are extremely sensitive to pollution, and their occurrence can be used to outline the extent of polluted areas [21,22]. Other species tolerate high levels of pollution, while some species even prefer metal-polluted sites [13,23]. Thus, undoubtedly, heavy metal content in soil is one of the most important edaphic factors determining bryophyte composition. We observed that Zn, Pb, and Cd concentrations in the soil, as well as the pollution load index, are important factors affecting species richness and cover of bryophytes. Moreover, the number of species proved to be significantly higher in the ‘extreme’ class than in the ‘low’ and ‘intermediate’ classes (Figure 3, Figure S1). Consequently, the sites characterized by high heavy metal concentrations in the soil and an alkaline pH could be considered as biodiversity hotspots for terrestrial bryophytes in a broad landscape scale of post-industrial areas. Contrarily, Rola and Osyczka [16] observed that in cryptogamic communities dominated by lichens, the species richness of cryptogams was lower in the plots characterized by higher pH values and high concentrations of toxic elements on the soil substrate. This indicated that the response to pollution could differ between taxonomic groups and/or were dependent on other external factors as well. Furthermore, Salemaa et al. [10] reported the decreasing abundance and species richness of both bryophytes and lichens along with pollution gradient, but the soil pH was strongly acidic in all the examined sites. This, in turn, indicated a significant impact of other soil factors on the pattern of changes in bryophyte diversity revealed in this study.

Soil pH was recognized as a factor that significantly affected the species richness of bryophytes in polluted sites (Table 2); however, its effect on bryophyte cover was not significant. Similarly, Becker and Brändel [24] observed that bryophyte species richness was strongly associated with both the copper content in soil and pH in a heavy metal–dry grassland complex in Germany. Dominant species from group C and most of the accompanying representatives of this group were strongly associated with basic and strongly basic substrates. Therefore, their occurrence in extremely polluted soils may be related to their preferences for an alkaline reaction (pH). Certainly, these species are able to withstand highly elevated levels of heavy metals, and thus should be regarded as tolerant, but not strictly limited, to metal-enriched substrates. It is also well reflected by the change in the share of species preferring alkaline soil according to their ecological indicator values along with increasing soil pollution (Figure 7). The remarkably high species richness of bryophytes was also reported in forest communities in the vicinity of an alkaline cement plant in Finland, which additionally emitted high levels of heavy metals [25]. This was explained by the abundant occurrence of calcicolous and pioneer species with a wide ecological amplitude near the pollution source. Furthermore, Paal and Degtjarenko [26] observed a high number of both epiphytic and epixylic bryophyte species in heavily polluted areas exposed to alkaline cement dust in Estonia. A similar phenomenon was also observed for plants. Vascular plant species richness and the proportion of metallophytes proved to be considerably higher in less acidic and calcareous wastes compared to acidic ones [27]. The pH level also determined that metal mobility in soils and toxic element availability were higher in acidic soils. Moreover, on calcareous substrates, the high content of Ca also reduced the toxicity of heavy metals [28]. This also applied to the studied plots located in grasslands that were developed in post-industrial wasteland consisting of dolomite and limestone as residues after Zn-Pb ore mining. All this can cause tolerant species to willingly inhabit polluted sites with alkaline soil. 

In addition to the chemical parameters of the soil, biotic interactions are also important drivers of bryophyte community structure. For example, Ingerpuu et al. [29] observed that the cover of two bryophyte species increased with the increasing vascular plant cover in experimental grassland plots. This indicated the beneficial effect of vascular plants by means of providing a better microclimate for bryophytes. On the other hand, the negative relationships between bryophyte species richness and cover of vascular plants were reported by Löbel et al. [30] in Swedish dry grasslands. This suggested the existence of competition between bryophytes and vascular plants. Interspecies competition is one of the processes affecting the richness of species on a local scale [31]. However, During and Lloret [32] suggested that competitive exclusion rarely occurs in bryophyte communities, which may not be solely due to the lack of competition, but results from a balance between competition and facilitation. We recognized vascular plant cover as a significant factor that negatively affected both the and species richness of bryophytes (Table 2). The abundance of bryophytes decreased with the increasing cover of the vascular plant layer. This can be explained by the competition between these two groups of plants [15], and this trend could additionally be enhanced by the high level of heavy metal pollution in the soil. Moreover, the intra- and inter-specific competition among bryophytes are not rare occurrences [33]. In particular, sometimes pleurocarpous mosses do not only behave as stress-tolerating strategists, and competition is one of the important factors for constructing their communities, and vegetation co-exists as a result of bryophytes and vascular plants. Nevertheless, bryophytes are more often weak competitors and they can simply be overrun by vascular plants, particularly in low-pollution sites [30], whereas high concentrations of heavy metals in combination with poorly developed soils certainly limit the occurrence and dispersal capabilities of vascular plants. Such a result shows that not only do abiotic habitat factors affect the richness and abundance of bryophytes, but the biotic aspect of the interaction with other organisms cannot be ignored. Finally, it can be concluded that the role of metal pollution in determining the species composition and richness of bryophyte communities is a complex phenomenon influenced by numerous abiotic and biotic factors. Consequently, the approach aimed at the recognition and evaluation of factors affecting these parameters cannot be limited to the analysis of simple relationships. 

Our results show that bryophyte species composition is clearly determined by soil chemical characteristics, mainly including heavy metal pollution levels and soil pH. The designated soil condition classes significantly differed in terms of species composition. The assessment of the heavy metal enrichment of a given soil based on terrestrial communities composed of both lichens and bryophytes has been previously reported [4,5,13]. Moreover, several indicator species for soils highly enriched with heavy metals has been recognized. These are known in the literature as ‘cooper mosses’, which are able to inhabit substrates with extremely high Cu contents, lethal to other mosses and liverworts, although some of them are associated with metals other than copper [34]. 

The bryophytes recorded in the present study are mainly common species often connected with anthropogenic habitats. Some of the species from group C, which were mainly recorded at polluted sites with an alkaline soil pH, were also reported in different heavy metal-polluted sites in Europe. *Weissia controversa* var. *densifolia* was recorded by Crundwell [35] in lead-mine wastes in the UK, whereas *Bryum pallescens* was growing in a abundance on old slags and wasteland in the region of Olkusz [36]. Both species were classified as facultative metallophytes indicative of Pb or Zn in Ireland [23]. *Tortella tortuosa* is a typical species confined to calcareous substrates, but is also characterized by a high level of heavy metal tolerance. It was also recorded in heavy metal-polluted sites in Greece [37], metalliferous grassland in the Harz Mountains [38], heavy metal–dry grassland complex in Germany [24], and in post-smelting dumps in southern Poland [39]. Zechmeister et al. [40] classified *Amblystegium serpens* and *Brachythecium rutabulum* as species insensitive to environmental pollution. Finally, *Bryum pseudotriquetrum* has been commonly used in biomonitoring studies to assess the environmental level of Zn and Pb pollution [6]. 

The species representing group B were recognized as bryophytes with a wide ecological tolerance range that covered a broad range of heavy metal content in soil. Among them are species that occur abundantly on various metalliferous substrates across Europe (e.g., *Brachythecium albicans*, *Ceratodon purpureus*, and *Pohlia nutans*; see [4,12,13,41,42]. Representatives of this group were also frequently recorded in artificial post-smelting dumps in the Upper Silesia region of Poland [43]. The production of special forms of vegetative reproduction and/or abundant sporophytes certainly helps them to successfully colonize such habitats. Zvereva and Kozlov [14] confirmed that bryophytes that possess various forms of vegetative reproduction (e.g., tubers, gemmae, and bulbils) were more tolerant to pollution than other species. The successful survival of species belonging to group B at disturbed sites is probably conditioned by their high reproductive capacity and rapid growth. Among these species, *Ceratodon purpureus* and *Pohlia nutans* were the most frequently observed. *Ceratodon purpureus* is considered pollution tolerant, connected with human disturbances, and often observed in man-made substrata or polluted soils [44]. This stress-tolerant ruderal species has been recognized as typical of metalliferous grasslands on mine wastes [41]. The abundance of *C. purpureus* was frequently higher in polluted sites than in unpolluted habitats [10,45]. *Pohlia nutans* not only survives under severe pollution impacts, but even colonizes heavily contaminated (barren) areas around non-ferrous smelters, being absent or rare in surrounding unpolluted habitats (e.g., [10,46,47]). These two species are primary pioneers colonizing areas after the slash burning of the forest [48]. The bare grounds without any vegetation near non-ferrous smelters may favor their establishment because they cannot grow in habitats with dense vascular field layer vegetation [49,50]. Their common occurrence in heavy metal-polluted sites is also associated with life strategy. They are classified into ‘colonists’ that live in sites where habitat start is unpredictable [51]. Post-mining wastes and grasslands developed near smelters examined in this study are typical representatives of such disturbed habitats. These two species gain additional advantages in polluted environments due to their high tolerance to prolonged dryness [52], which frequently occurs in post-industrial sites [50]. All these attributes could contribute to the success of these species in heavily polluted sites. 

The last group of species, represented by two typical species, i.e., *Cephaloziella divaricata* and *Polytrichum piliferum*, is considered as sensitive to heavy metal pollution and preferring acidic soil with relatively low contents of organic carbon and total nitrogen. The occurrence of species in group A is limited to low-polluted soils (Figure 5). Similarly, Rola and Osyczka [39] recorded that these species are only obtained from low-polluted, psammophilous grasslands among a wide variety of different habitats and post-industrial wastes polluted with heavy metals. Furthermore, Denayer et al. [13] classified *Polytrichum piliferum* in the group of bryophytes that only tolerate very low Pb and Cd concentrations in soil. 

As regards the indicator values for species in relation to their tolerance to heavy metal pollution [53], we decided not to include this parameter in our analysis. This was due to the fact that we recorded many bryophyte species that were classified, according to Hill et al. [53], in the group of species that are absent from substrates with moderate or high concentrations of heavy metals in Britain and Ireland. As many as six species representing group C and two species from group B were classified in this group, but readily occurred in Poland in extremely polluted sites, some of them at a high frequency, i.e., *Amblystegium serpens*, *Brachythecium salebrosum*, *Bryum caespiticium*, or *B. pseudotriquetrum*. This, nevertheless, indicated the significant differences in bryophyte communities in heavy metal-polluted sites in these two areas of Europe. On the other hand, the bryophyte assemblages were quite similar to those recorded in northern France in Zn-Pb polluted sites [4,13], since 12 bryophyte species were common to these two areas. Moreover, very similar changes in species composition along with changes in soil pH and level of heavy metal pollution were observed. Additionally, similar preferences of certain bryophyte species growing in different kinds of post-industrial dumps were recognized by Rola and Osyczka [5]. All this gives us reason to assume that bryophytes constitute good bioindicators not only in the context of heavy metal biomonitoring based on measurements of metal concentrations, but also at the community level concerning the changes in bryophyte community structure. However, when applying such monitoring methods, one must take into account the preferences of bryophyte species to soil pH. 

The bioindicative value of bryophytes can be considered in two different contexts, i.e., species turnover along the soil condition gradient and significant changes in species richness. Our study revealed a progressive replacement of bryophyte species along the soil pH and pollution gradient. This confirms that this group of organisms is highly responsive to environmental factors, and thus the changes in bryophyte composition may indicate the habitat conditions in a certain site. Similar observations were made in relation to bryophytes occurring in boreal forest communities [26]. These authors concluded that the simultaneous effect of heavy metal pollution and alkaline pH had a noticeable influence on species composition and the typological structure of bryophyte communities. The same issues concern bryophytes occurring in open, dry grasslands examined in this study, although the species composition was quite different (see Table 1 and [26]). A significant bryophyte species turnover along the magnesite pollution gradient from the most degraded open habitats to the visually unaffected forests was also revealed by Blanár et al. [54]. Additionally, two specialist species from strongly polluted sites were distinguished [54]. This proved that the community structure could be a good indicator of habitat conditions (see [55]). On the other hand, Degtjarenko et al. [56] concluded that the number of bryophyte species was a more promising indicator of environmental conditions than the occurrence of individual species under the influence of alkaline dust pollution emitted from limestone quarries. The authors observed a considerably higher number of bryophyte species near the pollution source, and recorded a gradual decrease in bryophyte richness along an increasing distance from the source of dust pollution [56]. Our results also show the significantly higher species richness of bryophytes in grassland communities on an alkaline substrate polluted with heavy metals. However, the remaining soil condition classes did not significantly differ among themselves, despite the fact that the pH value significantly decreased from the ‘high’ to ‘low’ class (Figure 2). Although we observed a significant positive relationship between bryophyte species richness and soil pH, we concluded that, in this case, the change in species richness itself could be a poor indicator of pollution level without considering the processes occurring in the structure of bryophyte communities and the replacement of certain species by others along an environmental gradient.

## 4. Materials and Methods

### 4.1. Study Area

The study was conducted in the area of the Olkusz Ore-bearing Region in the surroundings of a Zn smelter (‘ZGH Bolesław’ Mining and Smelting Works) in the Silesia-Cracow region (southern Poland). Zn-Pb ore-mining and smelting activities have contributed to the substantial metal pollution of soils in the study area [57]. The soils are mainly polluted by Zn, Pb, Cd, As, and Tl [58,59]. The study concerned the areas of dry grassland communities developed in post-mining waste and post-industrial sandy soils. According to the updated Köppen–Geiger climate classification [60], the study area is classified under a temperate oceanic climate (Cfb). Based on the data obtained from the local meteorological station, the mean annual temperature is 8.39 °C, mean annual precipitation sum amounts to 618.64 mm, and mean relative air humidity is 79.30% (calculated for 14 years of the 21st century, IMGW meteorological station—code ‘250190530′, data obtained from the Institute of Meteorology and Water Management, National Research Institute).

### 4.2. Field Studies, Sampling, and Bryophyte Identification

The fieldwork and sampling were conducted in the summer season of 2018. In total, 64 study plots, 1 m × 1 m, representing homogenous patches of vegetation, were examined with respect to the presence of bryophyte species. From each bryophyte population recorded in the plots, a sample was obtained for subsequent identification. Each sample was taken apart and studied in detail under a microscope to avoid overlooking any mixed species or even fragments of mosses and liverworts. Additionally, the coverage of bryophytes and vascular plants was estimated in each plot. It was estimated on a percentage scale within each study plot using digital photos of the vegetation (see [61]). The borders of each plot were marked with a colored cord; then a Nikon D5300 Digital Camera attached to a portable camera tripod was used to photograph the vegetation cover. The photos were taken from 1.5 m above the ground at a downward angle of 90° with the same field of view, resolution, and other settings. To ensure an exactly vertical position, a bubble level was used. Subsequently, the coverage was manually estimated by using Motic Images Plus 2.0 software (Hong Kong, China) and converted into a percentage of the plot surface (see [61]). The nomenclature of bryophytes follows Hill et al. [62]. Dried material in the form of herbarium specimens was deposited in an OSTR herbarium (University of Ostrava) for possible subsequent study. 

From each plot, three soil samples, from a depth of 5 cm, were collected and bulked in one composite sample, packed into paper bags, and transported to the lab.

### 4.3. Soil Chemical Analysis

Soil samples were dried and sieved (2 mm mesh). The soil pH was measured in air-dried samples in 1 M KCl suspensions with a Hach Lange HQ40d multimeter; organic carbon content was measured using a dry combustion method with an LECO SC-144DR Analyzer (LECO Corp., St. Joseph, MI, USA) and total nitrogen content using the Kjeldahl method with a Kjeltec 2300 Analyzer Unit (FOSS Tecator, Hoganas, Sweden). A total of 5 g DW soil samples was digested with 70% HClO_4_ (Merck, Suprapur) using a digester (FOSS Tecator 2020, Hoganas, Sweden). Subsequently, flame atomic absorption spectrometry was applied for the determination of Zn, Pb, Cd, and As concentrations (referred to herein as heavy metals) using a Varian 280 Fast Sequential Atomic Absorption Spectrometer (Varian, Melbourne, Australia). Certified reference material (CRM048-50G, sand, Sigma-Aldrich, St. Louis, MO, USA) was used for quality assurance; the recovery ranges were as follows: 99.2–99.9%, 97.3–98.8%, 97.9–99.8%, and 89.0–94.6% for Zn, Pb, Cd, and As, respectively.

### 4.4. Calculations and Statistical Analysis

The pollution load index (PLI) was calculated for the total assessment of the degree of soil contamination [63]. The PLI was calculated based on the following formula:

(1) $$PLI=\sqrt[{4}]{PIsoilZn\times PIsoilPb\times PIsoilCd\times PIsoilAs}$$

where PIsoil is a calculated value for a single pollution index calculated according to the following formula:

(2) $$PIsoil=\frac{Cnsoil}{Gbsoil}$$

where Cnsoil is the concentration of element in the soil sample and Gbsoil is the geochemical background according to Kabata-Pendias [64].

The cluster analysis based on the Bray–Curtis coefficient and the unweighted pair-group average (UPGMA) clustering algorithm was applied to compare the similarity of the study plots in terms of their soil chemical parameters. The similarity profile test (SIMPROF; [65]) was applied to confirm the significance of the designated groups. The procedure is a permutation test of the null hypothesis that a specified set of samples, which are not divided into groups a priori, contain no multivariate structure to examine further. The tests were performed on every node of a completed dendrogram. Then, non-metric multidimensional scaling (NMDS) was used for the same purposes [66]. Then, the study plots were classified into soil condition classes, based on the results of the aforementioned analyses.

The non-parametric Kruskal–Wallis test (*p* < 0.05), followed by post hoc Dunn’s test, was performed to verify the significance of differences in the soil parameters, bryophyte species richness, and total bryophyte cover (%) across the identified soil condition classes. A non-parametric test was applied since the homogeneity of variance assumption was not met (Brown–Forsythe test; *p* < 0.05). Principal component analysis (PCA) was performed to show the distribution of studied plots according to their soil chemical parameters in the form of data attribute plots (graphic forms) using the PCA function to show bryophyte species richness across all examined plots. The analysis was based on the correlation matrix.

Factor analysis based on the principal components was applied to obtain uncorrelated factors representing habitat parameters. The factors were extracted according to Cattell’s scree test [67] and varimax-rotated to facilitate their interpretation. Then, multiple effects of factors derived from the factor analysis of bryophyte species richness and cover were investigated through forward stepwise multiple linear regression analysis (with a threshold of F > 1.00 to entry). The analysis was applied to determine the factors that significantly affected species richness and the cover of bryophytes, and to designate the dominant factor influencing bryophyte traits. The procedure constituted a combination of the forward selection and backward elimination. The initial models included only a regression constant and a predictor with the highest input statistic (F-to-enter) was firstly entered into the model. Prior to the analysis, the following assumptions were verified in order to validate the models: distribution normality of residuals were checked using the Kolmogorov–Smirnov test (*p* > 0.05), the potential multicollinearity of the predictors was verified by calculating the variance inflation factors (VIFs), and Durbin–Watson statistics were calculated to assess the potential presence of a serial correlation of residuals. A detailed residual analysis was performed to detect the potential outliers and/or influential points.

Permutational multivariate analysis of variance (PERMANOVA) was performed to test for the differences in bryophyte species composition between plots representing different soil condition classes [68]. Pair-wise comparisons among all pairs of classes were calculated as multivariate pseudo-t statistics and *p*-values were obtained using a permutation procedure along with average resemblances between classes. Due to the unbalanced design, type III SS was used for partitioning. The analyses were based on the matrix of the presence/absence of bryophyte species using the Jaccard coefficient, with 999 permutations for each test. Prior to the analysis, the homogeneity of multivariate dispersions (PERMDISP routine) was applied to test the relative within-group dispersions among the groups. To determine the statistical correlation between the two similarity matrices of the study plots, the Mantel test was applied. The first matrix was calculated based on the soil chemical parameters and the second on binary data concerning the presence/absence of particular species in the plots. Bray–Curtis and Jaccard coefficients were applied to the first and second matrices, respectively. This was performed in order to determine whether between-plot similarities in terms of soil chemistry and species presence/absence were significantly interrelated.

The seriation of all bryophyte species recorded in the study plots was conducted using a constrained algorithm [69]. The plots were arranged in accordance with soil condition classes. The seriation procedure attempted to re-organize the presence of species to be concentrated diagonally, revealing the gradient of changes in species composition along with changing soil conditions. Non-metric multidimensional scaling (NMDS) was applied to obtain the pattern of similarities in the occurrences of species in the studied plots. The Jaccard coefficient was used. Based on the above-mentioned analyses, bryophyte species were classified into three groups of species reflecting the similar patterns of their occurrences depending on soil conditions. 

The proportion of bryophyte occurrence with the selected ranges of ecological indicator values, i.e., light (L), moisture (M), pH reaction ^®^, and nitrogen (eutrophication, N), in particular soil condition classes was calculated. The ecological indicator values were assigned to particular species according to Ellenberg et al. [70] and modified by Hill et al.’s [54] classification. The ranges of ecological indicator values were as follows: light (4–5, 6–7, 8–9), moisture (1–3, 4–5, 6–7, 8–9), reaction (2–3, 4–5, 6–7, 8), nitrogen (1–2, 3–4, 5–7). Subsequently, the significance of differences between soil condition classes was verified using non-parametric Kruskal–Wallis test (*p* < 0.05). Dunn’s tests were applied for post hoc comparisons. The analysis was performed only in the cases where all soil condition classes were represented by the given group of species classified into the range of indicator values (i.e., variance was present within each soil condition class).

The Statistical analyses were performed using PRIMER 7 statistical software (Primer-E, Plymouth UK; [71]), PAST 3.25 [72], Statgraphics Centurion 18 (Statgraphics Technologies, Inc., The Plains, VA, USA), and STATISTICA 13 (TIBCO Software Inc., Palo Alto, CA, USA).

## 5. Conclusions

The results show that bryophyte communities highly depend on soil heavy metal pollution levels. Soil pH was recognized as the second most important factor that affected bryophyte species richness in polluted sites. The most symptomatic was a clear change in bryophyte species composition along with increasing concentrations of heavy metals and soil pH. This proved that bryophytes were highly responsive to soil factors, and thus the changes in bryophyte community structure may indicate the habitat conditions of a certain site. Furthermore, bryophyte species richness increased along with increasing concentrations of heavy metals on the soil substrate. Consequently, the sites characterized by an alkaline pH and high heavy metal concentrations could be considered as biodiversity hotspots for terrestrial bryophytes in post-industrial landscapes. Apart from soil characteristics, biotic interactions were also recognized as important drivers of bryophyte communities. Bryophyte species richness and abundance decreased with the increasing cover of the vascular plant layer. Such results show that the effect of metal pollution on bryophyte community structure is a complex phenomenon that is additionally influenced by both abiotic and biotic factors. Finally, the bioindicative value of bryophytes could be considered in two different contexts: species turnover along the soil pH and pollution gradient, and significant changes in species richness. However, we concluded that the number of bryophyte species itself could be an insufficient indicator of the soil pollution level without considering the processes occurring in the structure of bryophyte communities in relation to the replacement of certain species by others along with gradient of changing soil conditions.

## Figures and Tables

**Table 1:** Factors derived from habitat properties, i.e., soil chemical parameters and vascular plant cover. Factor loadings are given in parentheses; only variables with factor loadings greater than 0.7 are listed. The percentage of explained variance for each factor is provided.

Factor No.	Variables (Factor Loadings)	Variance Explained (%)
Factor 1	Vascular plant cover (−0.91)	53.81
Factor 2	C organic (0.97), N total (0.96)	21.85
Factor 3	Pb (0.94), PLI (0.83), Cd (0.72), Zn (0.70)	7.99
Factor 4	pH (0.92)	7.02

**Table 2:** Result of forward stepwise multiple regression analysis for the effect of factors related to habitat parameters on bryophyte species richness (R^2^ = 0.45, F = 14.97, *p* < 0.05) and bryophyte cover (R^2^ = 0.47, F = 17.70, *p* < 0.05). For a description of the factors, see Table 1. Variables with a significant effect (*p* < 0.05) are provided in bold. The variables are listed according to the *p*-value.

Bryophyte Species Richness				
N = 62	Standardized β Coefficient	SE for β Coefficient	*t*	*p*
Constant			**16.466**	**<0.001**
Factor 1	0.486	0.100	**4.849**	**<0.001**
Factor 3	0.450	0.100	**4.509**	**<0.001**
Factor 4	0.261	0.100	**2.606**	**0.012**
**Bryophyte Cover**				
**N = 64**	**Standardized β Coefficient**	**SE for β Coefficient**	***t***	***p***
Constant			**13.230**	**<0.001**
Factor 1	0.625	0.094	**6.656**	**<0.001**
Factor 3	0.220	0.094	**2.340**	**0.023**
Factor 4	0.179	0.094	**1.905**	**0.062**

**Table 3:** PERMANOVA pairwise comparisons of bryophyte species composition between soil condition classes. Lower diagonal—*p*-values by permutation. Upper diagonal—average similarities between groups.

Soil Condition Class	Low	Intermediate	High	Extreme
Low		29.63	7.46	9.85
Intermediate	0.003		8.77	10.62
High	0.001	0.001		21.42
Extreme	0.001	0.001	0.004	

**Table 4:** Defined three groups of bryophytes (A, B, and C) according to their sensitivity to heavy metal pollution.

**GROUP A**—species sensitive to heavy metal pollution, growing on acidic soil with a relatively low content of organic carbon and total nitrogen	**GROUP B**—nonspecific species tolerant to elevated heavy metal concentration in soil, but inhabiting sites with a wide spectrum of heavy metal concentrations in soil	**GROUP C**—species preferring heavy metal-polluted soils with high organic carbon and total nitrogen contents and slightly alkaline pH
**Frequent**	**Frequent**	**Frequent**
*Cephaloziella divaricata*	*Ceratodon purpureus*	*Tortella tortuosa*
*Polytrichum piliferum*	*Bryum caespiticium*	*Amblystegium serpens*
	*Pohlia nutans*	*Campylium chrysophyllum*
	*Barbula unguiculata*	*Weissia controversa*
		*Bryum pseudotriquetrum*
		*Brachythecium salebrosum*
		*Calliergonella cuspidata*
**Additional**	**Additional**	**Additional**
*Polytrichum juniperinum*	*Bryum argenteum*	*Schistidium crassipilum*
*Brachythecium velutinum*	*Hypnum cupressiforme*	*Plagiomnium affine*
*Cirriphyllum piliferum*	*Brachythecium albicans*	*Plagiomnium undulatum*
		*Rhizomnium punctatum*
		*Bryum pallescens*
		*Eurhynchium hians*
		*Plagiomnium cuspidatum*
		*Sanionia uncinate*
		*Abietinella abietina*
		*Brachythecium rutabulum*
		*Homalothecium lutescens*
		*Thuidium recognitum*
		*Eurhynchium praelongum*

## Data Availability

The data presented in this study are available in the article and the Supplementary Material.

## Supplementary Materials

The following supporting information can be downloaded at: https://www.mdpi.com/article/10.3390/plants11162091/s1, Figure S1: Principal component analysis (PCA) ordination diagram based on soil chemical parameters in the study plots representing different soil condition classes in a form of data attribute plots of bryophyte species richness recorded in the same plots. Increasing size of the symbols indicates an increase in the number of species. The percentage of variance explained by the principal components are provided in parentheses. For detailed characteristics of soil condition classes, see Figure 2; Table S1: Results of Kruskal–Wallis tests (*p* < 0.05) for significance of differences between soil condition classes in terms of the proportion of bryophyte species classified in selected ranges of ecological indicator values [53,70]. The analysis was performed only in cases when variance was present within each soil condition class. Significant differences are provided in bold (*p* < 0.05).
