# Supplementary material for: The Utility of Ground Bryophytes in the Assessment of Soil Condition in Heavy Metal-Polluted Grasslands
Source: Plants (Basel). 2022 Aug 11;11(16):2091. doi: 10.3390/plants11162091 (PMC9416651; doi:10.3390/plants11162091)
Supplement: Supplementary file 1 [file plants-11-02091-s001.zip › plants-1858451-supplementary.pdf]

The Utility of Ground Bryophytes in the Assessment of Soil Condition in Heavy Metal-Polluted Grasslands

Kaja Rola [1] and Vítězslav Plášek [2,3,*]

[1] Institute of Botany, Faculty of Biology, Jagiellonian University, Gronostajowa 3, 30-387 Kraków, Poland;
kaja.skubala@uj.edu.pl
[2] Department of Biology and Ecology, Faculty of Science, University of Ostrava, Chittussiho 10,
CZ-710 00 Ostrava, Czech Republic
[3] Institute of Biology, University of Opole, Oleska 22, 45-052 Opole, Poland
* Correspondence: vitezslav.plasek@osu.cz

Table S1. Results of Kruskal-Wallis tests ($p < 0.05$) for significance of differences between soil condition classes in terms of the proportion of bryophyte species classified in selected ranges of ecological indicator values (Ellenberg et al. (1991) [70] and modified by Hill et al. (2007) [53] classification). The analysis was performed only in cases when variance was present within each soil condition class. Significant differences are provided in bold ($p < 0.05$).

Ecological indicator values	H	p
Light values (L)		
4-5	4.65	0.199
6-7	1.68	0.642
8-9	6.53	0.088
Moisture values (F)		
1-3	7.80	0.051
4-5	**14.69**	**0.002**
6-7	-	-
8-9	-	-
Reaction (R)		
2-3	-	-
4-5	**22.39**	**< 0.001**
6-7	**34.31**	**< 0.001**
8	-	-
Nitrogen (N)		
1-2	**9.20**	**0.027**
3-4	6.18	0.103
5-7	**12.17**	**0.007**

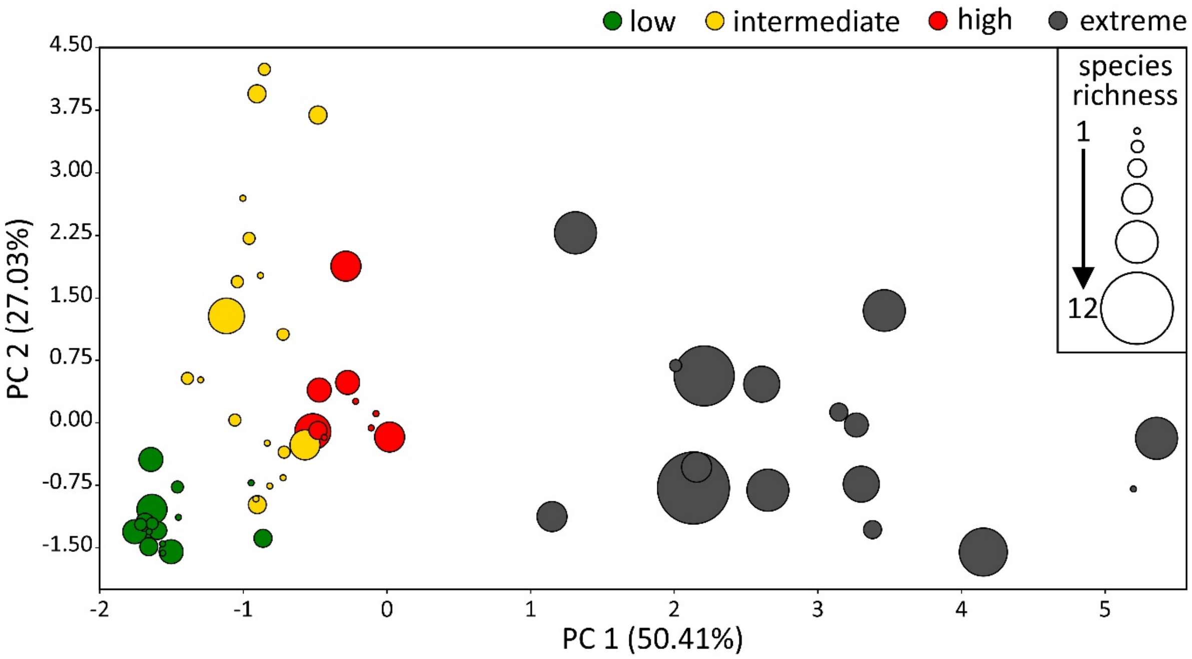

Figure S1. Principal component analysis (PCA) ordination diagram based on soil chemical parameters in the study plots representing different soil condition classes in a form of data attribute plot of bryophyte species richness recorded in the same plots. Increasing size of the symbols indicates an increase in the number of species. The percentage of variance explained by the Principal Components are provided in parentheses. For detailed characteristics of soil condition classes see Figure 2.
